# Design and Identification of a Novel Antiviral Affinity Peptide against Fowl Adenovirus Serotype 4 (FAdV-4) by Targeting Fiber2 Protein

**DOI:** 10.3390/v15040821

**Published:** 2023-03-23

**Authors:** Xiao Chen, Qiang Wei, Fusheng Si, Fangyu Wang, Qingxia Lu, Zhenhua Guo, Yongxiao Chai, Rongfang Zhu, Guangxu Xing, Qianyue Jin, Gaiping Zhang

**Affiliations:** 1 College of Veterinary Medicine, Northwest A&F University, Xianyang 712100, China; 2 Henan Provincial Key Laboratory of Animal Immunology, Henan Academy of Agricultural Sciences, Zhengzhou 450002, China; 3 Institute of Animal Science and Veterinary Medicine, Shanghai Academy of Agricultural Sciences, Shanghai 201106, China; 4 College of Life Science, Henan Agricultural University, Zhengzhou 450002, China; 5 Jiangsu Co-Innovation Center for Prevention and Control of Important Animal Infectious Diseases and Zoonoses, Yangzhou University, Yangzhou 225009, China

**Keywords:** fowl adenovirus 4 (FAdV-4), Fiber2 protein, crystal structure, virtual screening, antiviral peptide

## Abstract

Outbreaks of hydropericardium hepatitis syndrome caused by fowl adenovirus serotype 4 (FAdV-4) with a novel genotype have been reported in China since 2015, with significant economic losses to the poultry industry. Fiber2 is one of the important structural proteins on FAdV-4 virions. In this study, the C-terminal knob domain of the FAdV-4 Fiber2 protein was expressed and purified, and its trimer structure (PDB ID: 7W83) was determined for the first time. A series of affinity peptides targeting the knob domain of the Fiber2 protein were designed and synthesized on the basis of the crystal structure using computer virtual screening technology. A total of eight peptides were screened using an immunoperoxidase monolayer assay and RT-qPCR, and they exhibited strong binding affinities to the knob domain of the FAdV-4 Fiber2 protein in a surface plasmon resonance assay. Treatment with peptide number 15 (P15; WWHEKE) at different concentrations (10, 25, and 50 μM) significantly reduced the expression level of the Fiber2 protein and the viral titer during FAdV-4 infection. P15 was found to be an optimal peptide with antiviral activity against FAdV-4 in vitro with no cytotoxic effect on LMH cells up to 200 μM. This study led to the identification of a class of affinity peptides designed using computer virtual screening technology that targeted the knob domain of the FAdV-4 Fiber2 protein and may be developed as a novel potential and effective antiviral strategy in the prevention and control of FAdV-4.

## 1. Introduction

Fowl adenoviruses (FAdVs), belonging to the *Aviadenovirus* genus of the *Adenoviridae* family, are grouped into 5 species (FAdV-A to -E) and further divided into 12 serotypes (FAdV-1 to 8a and 8b to 11) on the basis of profiles of restriction enzyme digestion and serum cross-neutralization assays [1,2]. Hepatitis-Hydropericardium syndrome (HHS) associated with fowl adenovirus serotype 4 (FAdV-4) infection was first reported in Angara Goth, Pakistan, in 1987 and is known as Angara disease [3]. HHS subsequently spread to other Asian countries, where it became endemic, but also to some Arabian countries and parts of Latin America [4], including reports from the Middle East [5], Russia [6], Slovakia [7], Central and South America [8,9], Japan [10], and Korea [11]. HHS has been commonly reported to be caused by FAdV-4 in China and has been associated with high mortality rates since 2015 [12,13,14,15,16]. The causative agent for HHS was identified as the highly virulent Chinese FAdV-4 isolate with a large genomic deletion (1966 bp) between *ORF42* and *ORF43* [17,18]. FAdV-4 has a wide range of hosts, including laying hens, broilers [19], ducks [20], and geese [21]. FAdV-4 is transmitted not only horizontally through the fecal–oral route but also vertically through chicken embryos, making it highly contagious [22,23].

Emerging and re-emerging viral infections attract worldwide attention and greatly promote the development of antiviral agents. Peptides with antiviral activity stand out because of their biocompatibility, specificity, and effectiveness, and because they overcome the limitations of existing drugs [24]. In particular, synthetic peptides designed using virtual screening technology and molecular docking technology could shorten the research cycle and costs and significantly increase the possibility of screening for desirable results [25,26]. The technology of molecular docking has been widely used to find suitable therapeutic targets or drugs against various diseases, such as COVID-19 [27,28], AIDS [29], and Dengue fever [30]. Computer virtual screening technology that relies on molecular docking technology can simulate the binding of peptide ligands and proteins, which have known crystal structures; predict the structure of a ligand within the constraints of a receptor binding site; and correctly estimate the strength of binding using a scoring function [31]. Thus, this study used virtual screening and molecular docking technology to design affinity peptides that accurately target proteins and explored their application in antiviral assays.

FAdV-4 encodes 10 major structural proteins, including hexon; penton base; fiber-1; fiber-2; terminal protein; and proteins VI, VII, VIII, III, and μ [22]. Several recent studies have reported that the pathogenicity of the emerging highly pathogenic FAdV-4 is closely related to the *fiber2* gene [32,33]. The Fiber2-protein-based subunit vaccine offered excellent protection against a FAdV-4 challenge [34,35,36,37,38], revealing that Fiber2 might be an efficient target for developing vaccine candidates against FAdV-4. Furthermore, a novel monoclonal antibody (mAb), 3C2, that targeted Fiber2 efficiently blocked FAdV-4 infection, which provides strong evidence that the Fiber2 protein plays vital roles in mediating FAdV-4 infection [39]. FAdV-4 antiviral agents targeting Fiber2 may play an important antiviral role. Moreover, the C-terminal knob domain of the FAdV-4 Fiber2 protein is exposed on the outer surface, which makes interacting with other molecules easier. In this study, the crystal structure of the C-terminal knob domain of the FAdV-4 Fiber2 protein was determined for the first time. Furthermore, a series of affinity peptides targeting the knob domain of the Fiber2 protein were designed on the basis of the crystal structure using computer virtual screening technology. Eventually, peptide number 15 (P15) was found to be an optimal peptide with antiviral activity against FAdV-4 in vitro, laying a foundation for future applications in the prevention and control of FAdV-4 infections.

## 2. Materials and Methods 

### 2.1. Cells and Virus Strain

Leghorn male hepatocellular (LMH) cells were cultured in Dulbecco’s modified Eagle’s medium (DMEM) (Sigma-Aldrich, Saint Louis, MI, USA) supplemented with 10% fetal bovine serum (FBS) (Gibco, ThermoFisher Scientific, Waltham, MA, USA), 100 IU/mL penicillin, and 100 μg/mL streptomycin in a 37 °C incubator supplied with 5% CO_2_. The FAdV-4 strain ZZ (GenBank accession no. MN337322.1) was identified and preserved in our laboratory.

### 2.2. Reagents and Antibodies

His-tag monoclonal antibody was purchased from Proteintech (Wuhan, China). The rabbit polyclonal antibody and the mAb 5A5 against the FAdV-4 Fiber2 protein were prepared in our laboratory. The affinity peptides were screened with molecular docking in our laboratory using SYBYL-X 2.1.1 software (Tripos, St. Louis, MI, USA) and then synthesized by GL Biochem Ltd. (Shanghai, China). CM5 chips were purchased from GE (General Electric Company, Fairfield, CT, USA). The cell counting kit-8 (CCK-8) kit was purchased from Beyotime (Shanghai, China). Positive chicken serum against FAdV-4 was prepared in our laboratory.

### 2.3. Expression and Purification of the Knob Domain of the Fiber2 Protein

The knob domain of the FAdV-4 Fiber2 protein was determined to be aa279-479 through homology comparison and modeling on the basis of the existing crystal structure of the adenovirus fiber protein. The knob domain of the FAdV-4 Fiber2 protein (amino acids 279–479) was expressed in *E. coli* BL21(DE3) cells using induction with a final concentration of 0.1 mM isopropyl-β-D-thiogalactoside (IPTG) overnight at 16 °C. The preliminary purification of the protein was performed using a Ni–NTA column (Merck Millipore, Darmstadt, Germany) and further purified using a gel-filtration column, Superdex200 Increase 10/300 GL (GE, Fairfield, CT, USA). The purity and bioactivity of the protein were determined with 12.5% sodium dodecyl sulfate–polyacrylamide gel electrophoresis (SDS–PAGE) and Western blot with a His-tag monoclonal antibody (dilution 1:5000).

### 2.4. Crystallization, Data Collection, and Structural Determination of Fiber2

Crystallization of the knob domain of the FAdV-4 Fiber2 protein was carried out at room temperature (25 °C) using the sitting-drop vapor-diffusion method with an equal volume of the target protein at 15 mg/mL and various crystallization reagents from crystallization screening kits (Hampton). The crystal picture of the FAdV-4 Fiber2 knob has been shown in Figure S1. The crystals were flash-frozen in liquid nitrogen using a cryoprotection solution with 20% glycerol in the crystallization solution. X-ray data sets of the crystals were collected at a wavelength of 0.97 Å and a temperature of 100 K on the beamline BL19U1 at the Shanghai Synchrotron Radiation Facility (SSRF). All data sets were processed using HKL-2000 [40]. The structure was determined with molecular replacement using Phaser [41] with the structure of a chicken embryo lethal orphan (CELO) short-fiber knob (PDB ID: 2VTW) as the search model. The structure was refined using phenix.refine [42] and manually adjusted with Coot [43]. The atomic coordinates and structure factors of the knob domain of the FAdV-4 Fiber2 protein have been deposited in the Protein Data Bank (PDB ID: 7W83). The statistics of data collection and refinement are summarized in Table 1.

### 2.5. Analytical Ultracentrifugation

Sedimentation velocity experiments were performed in a Beckman Coulter ProteomeLab XL-A analytical ultracentrifuge (Beckman Coulter Inc., Brea, CA, USA) at 25.0 °C and 50,000 rpm in standard two-sector cells using an An-60 Ti rotor. Samples were equilibrated in the rotor at 25.0 °C for at least 1 h prior to the collection of 144 scans over a 2.5 h period. Initial analyses were performed in SEDFIT (60) using a continuous *c(s)* model with a resolution of 120 and S ranging from 0 to 15.

### 2.6. The Design and Virtual Screen of the Peptides

The design and screening methods of all the peptides were described in the previous articles [44,45]. The original docking peptide conformation was designed using the Biopolymer/Build/Build Protein module in SYBYL-X 2.1.1 software on the basis of the crystal structure of the C-terminal knob domain of the FAdV-4 Fiber2 protein. Then, a peptide library consisting of a series of random peptides with different lengths (2–9 amino acids) was designed after hydrogenation, MMFF94 charge addition, and energy gradient optimization. The best active pocket region was selected by analyzing the crystal structure of the knob domain of the Fiber2 protein of FAdV-4 docked with the polypeptide library using the Surflex-Dock program in the SYBYL-X 2.1.1 software. Finally, the docking results were evaluated and analyzed comprehensively using a software scoring system. The affinity peptides were synthesized on the basis of standard Fmoc solid-phase peptide synthesis (Fmoc-SPPS) and purified using reversed-phase high-performance liquid chromatography (RP-HPLC). The syntheses of the affinity peptides were executed by GL Biochem Ltd. (Shanghai, China).

### 2.7. Immunoperoxidase Monolayer Assay

FAdV-4 virus, at a multiplicity of infection (MOI) of 0.01, was premixed with 200 μM peptides at 37 °C for 1 h and then inoculated onto near-confluent LMH cell monolayers at 37 °C in 5% CO_2_. The virus inoculum was removed after 1 h, and the infected cells were maintained in DMEM with 2% FBS for 24 h. After removing the supernatant, the cells were washed twice with PBS and fixed with ice-cold methanol at –20 °C for 30 min. The plates were then blocked with 5% skim milk at 37 °C for 1 h, followed by incubation with positive serum against FAdV-4 at 37 °C for 1 h and HRP-conjugated rabbit anti-chicken IgG (H + L) at 37 °C for 40 min. During each step, the plates were washed with PBST six times. The results were obtained using an AEC Peroxidase Substrate kit (Solarbio) and observed with an inverted microscope.

### 2.8. Real-Time Quantitative Polymerase Chain Reaction

The FAdV-4 copy number was determined using a real-time quantitative polymerase chain reaction (RT-qPCR). Viral DNA was extracted from the cells with a TaKaRa Mini Viral RNA/DNA Extraction kit Version 5.0 (TaKaRa Biotechnology Co., Ltd., Dalian, China) according to the manufacturer’s protocols. The FAdV-4 *Hexon* gene was used as an indicator for the presence of viral DNA. The standard positive plasmid was serially diluted 10-fold to 10^–2^–10^–8^ to generate the standard curve, and the copy numbers of the samples were quantified using the absolute quantification method. The primers for the RT-qPCR were designed using Primer 5.0 software (Table 2) and synthesized by Sangon Biotech (Shanghai, China). The RT-qPCR was performed on an Applied Biosystem 7500 Fast instrument under the following cycling conditions: 95 °C (10 min) and 40 cycles at 95 °C (10 s) and 60 °C (30 s). The standard curves were generated, and the quantity of the viral DNA in the samples was calculated.

### 2.9. Surface Plasmon Resonance Assay

The equilibrium dissociation constant (KD) of each peptide was determined using a Biacore X100 instrument (General Electric Company, Fairfield, CT, USA). All the experimental procedures were performed in accordance with the Biacore X100 manual. The knob domain of the FAdV-4 Fiber2 protein was covalently coupled to a CM5 chip using the EDC/NHS method at the optimal pH, which was determined first in a pre-experiment. The running buffer (HBS-EP, pH 7.4, filtered with a 0.22 μm filter) was then allowed to flow through the CM5 chip. The peptides to be detected were diluted with HBS-EP in 2-fold serial dilutions and then injected into the machine from a low concentration to a high concentration to observe the response signal changes in real time. In each cycle, the peptide solution was set to flow through the chip for 120 s at a constant flow rate of 30 μL/min, and then HBS-EP was injected to flow through the chip for 120 s to dissociate the peptides from the protein. Then, 0.25% SDS was used to completely elute the peptides. Ultimately, the kinetic dissociation constants of the binding reactions were calculated and analyzed using Biacore X100 Evaluation software, version 2.0.2 (General Electric Company, Fairfield, CT, USA).

### 2.10. Cytotoxicity Assay

The cytotoxicity of the peptides in the LMH cells was assayed using the CCK-8 method. The LMH cells were seeded in 96-well plates with a density of 5 × 10^3^ cells/well at 37 °C in 5% CO_2_. When the LMH cells formed a monolayer, the medium was discarded and replaced with FBS-free DMEM containing different concentrations of a peptide (0, 1.56, 3.125, 6.25, 12.5, 25, 50, 100, and 200 μM). Then, 10 μL of CCK-8 reagent was added to each well after 24, 48, and 72 h of incubation and then further incubated at 37 °C for 1 h. The absorbance of each well at 450 nm was read with a microplate analyzer.

### 2.11. TCID_50_ and Indirect Immunofluorescence Assay

FAdV-4 virus (MOI = 0.01) was mixed with different concentrations of peptide at 37 °C for 1 h and then inoculated into near-confluent LMH cell monolayers at 37 °C in 5% CO_2_. The virus inoculum was removed after 2 h, and the infected cells were maintained in DMEM with 2% FBS. The virus infectivity was assessed by measuring the 50% tissue culture infectious dose (TCID_50_) at various time points. The cytopathic effects (CPEs) were observed under the microscope, and the values of TCID_50_ were calculated following the Reed–Muench method. Meanwhile, the virions in the infected cells were detected with IFAs at 24 h.p.i. IFAs were performed with the mAb 5A5 prepared in our laboratory (1:500 dilution) as the primary antibody and FITC-conjugated goat anti-mouse IgG as the secondary antibody. The cell nuclei were stained with 4′, 6-diamidino-2-phenylindole (DAPI, Beyotime, Shanghai, China) for 5 min in the dark. The samples were observed with an inverted fluorescence microscope (Olympus, Tokyo, Japan). The expression level of the FAdV-4 Fiber2 protein was measured with Western blot using rabbit polyclonal antibodies against the FAdV-4 Fiber2 protein, and the results were visualized with ECL reagent. Images were obtained with a chemiluminescence imaging system (Fusion FX7; VILBER, Paris, France).

### 2.12. Statistical Analysis

All the statistical analyses were performed using GraphPad Prism version 8.0 (GraphPad Software, San Diego, CA, USA). Three replicates were included in each experiment, and each experiment was independently repeated at least three times. All the statistical analyses and calculations are expressed as mean ± SEM. Statistical significance was determined with an unpaired *t* test when only two groups were compared or with a one-way analysis of variance (ANOVA) when more than two groups were compared. Statistical significance was determined at the levels of *p* < 0.05 (*), *p* < 0.01 (**), *p* < 0.001 (***), or *p* < 0.0001 (****).

## 3. Results

### 3.1. Expression and Purification of Recombinant Protein Fiber2

**Figure 1:**
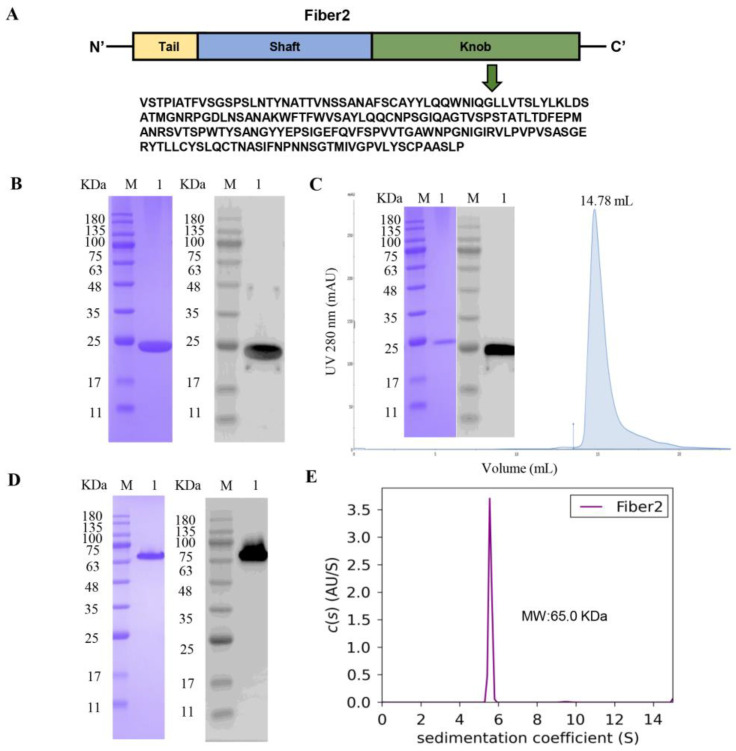
Characterization of the knob domain of the FAdV-4 Fiber2 protein. The knob domain of the FAdV-4 Fiber2 protein was expressed in *E. coli* BL21(DE3). (**A**) A schematic of the Fiber2 protein and the sequence expressed. (**B**) SDS–PAGE (left) and Western blot (right) analysis of purified knob domain of Fiber2 protein with Ni–NTA column. Lane M: molecular weight markers; Lane 1: purified protein. (**C**) The knob domain of Fiber2 was further purified with a Superdex200 Increase 10/300 GL and identified using SDS–PAGE (left) and Western blot (right). (**D**) SDS–PAGE (left) and Western blot (right) analyses of the protein without boiling. A His-tag monoclonal antibody was used in Western blot assays. (**E**) Analytical ultracentrifugation (AUC) analysis of the knob domain of Fiber2. The purple curve is representative of the SEDFIT software *c(s)* distributions of the knob domain of the FAdV-4 Fiber2 protein.

The knob domain of the FAdV-4 Fiber2 protein was expressed in *E. coli* BL21(DE3) cells and purified with a Ni–NTA column. A schematic of the Fiber2 protein is shown in Figure 1A. The SDS–PAGE and Western blot results show that the protein was purified with an apparent molecular mass of 23 kDa (Figure 1B). The purified protein was further eluted at 14.78 mL using a Superdex200 Increase 10/300 GL (Figure 1C), which suggests that the protein might exist as a trimer. Moreover, the protein that was not boiled before SDS–PAGE appeared at 65 kDa, demonstrating that the protein was trimeric in the elution (Figure 1D). The result of sedimentation velocity analytical ultracentrifugation (AUC) further confirms that the molecular weight of the protein was about 65.0 kDa in solution, revealing the native trimeric form of the protein (Figure 1E).

### 3.2. Crystal Structure of the Knob Domain of FAdV-4 Fiber2

**Figure 2:**
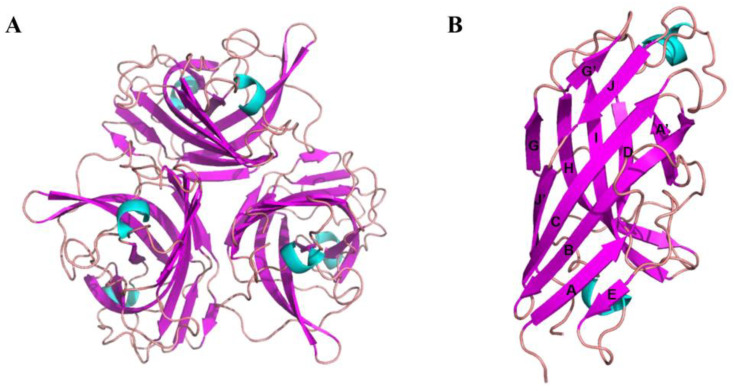
Structure of the knob domain of the FAdV-4 Fiber2 protein. (**A**) Ribbon representation of the trimer of the FAdV-4 Fiber2 knob (top view). The structure is depicted with the β-strands colored in magenta, α-helixes in cyan, and loops in pink. (**B**) Ribbon representation of a monomer of the FAdV-4 Fiber2 knob (side view). The β-strands were labeled according to the nomenclature introduced by Xia et al. [46].

Optimal crystals were acquired with a reservoir solution containing 0.1 M sodium acetate trihydrate, pH 7.0, and 12% *w*/*v* polyethylene glycol 3350. The crystal structure was solved with molecular replacement using a monomer of the knob domain of the CELO short fiber as a search model (PDB ID: 2VTW). The asymmetric unit contained two monomers, which formed two trimers by generating a symmetry mate (Figure 2A). Each monomer formed an anti-parallel β-sandwich with a topology similar to other known structures of knob domains of adenovirus fiber protein. The β-sandwich comprised two β-sheets, ABCJ and GHID (Figure 2B), for which nomenclature was proposed for the knob domain of the HAdV5 fiber [46].

### 3.3. Design and Synthesis of Peptides on the Basis of the Crystal Structure of the Knob Domain of the FAdV-4 Fiber2

**Figure 3:**
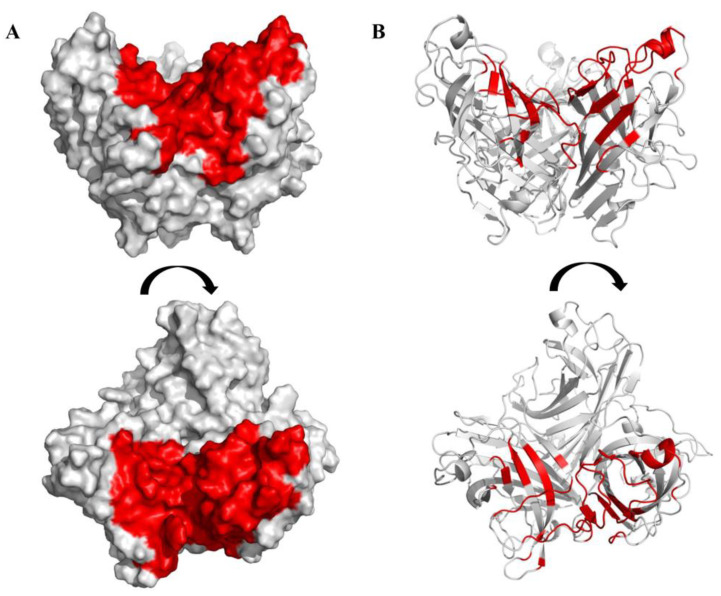
The docking active pocket of the C-terminal knob domain of the FAdV-4 Fiber2 protein. The C-terminal knob domain of the FAdV-4 Fiber2 protein selected as the docking active pocket is displayed in surface (**A**) and cartoon (**B**) images, with the side view (top) and top view (bottom). The docking active pocket is colored in red. The trimeric protein is colored in gray.

A region of the knob domain of the FAdV-4 Fiber2 protein comprising 81 amino acids was selected as the docking active pocket on the basis of the structure obtained above (Figure 3). The docking active pocket included the top of the trimer and the region between the two chains (sequences are shown in Figure S2). A virtual library of linear peptides with a capacity of 24,000 was obtained after hydrogenation, MMFF94 charge addition, and energy gradient optimization. After molecular docking between the selected docking pocket and the peptide library with the Surflex-Dock program using SYBYL-X 2.1.1 software, the affinity between the peptides and the knob domain of the FAdV-4 Fiber2 protein were evaluated using the total score values, which were calculated with a consensus score function. Subsequently, 30 peptides with higher total score values were synthesized for subsequent experiments (Table 3).

### 3.4. Screening of Peptides

Each of the 30 peptides was added to the LMH cells at a concentration of 200 μM for 24 h, and the morphology of the LMH cells was examined under a microscope as a preliminary evaluation of the cytotoxicity of each peptide. The results show that nine peptides (P3, P8, P9, P11, P12, P18, P19, P23, and P29) damaged cells severely, causing the cells to shrink and even break into fragments (Figure S3).

**Figure 4:**
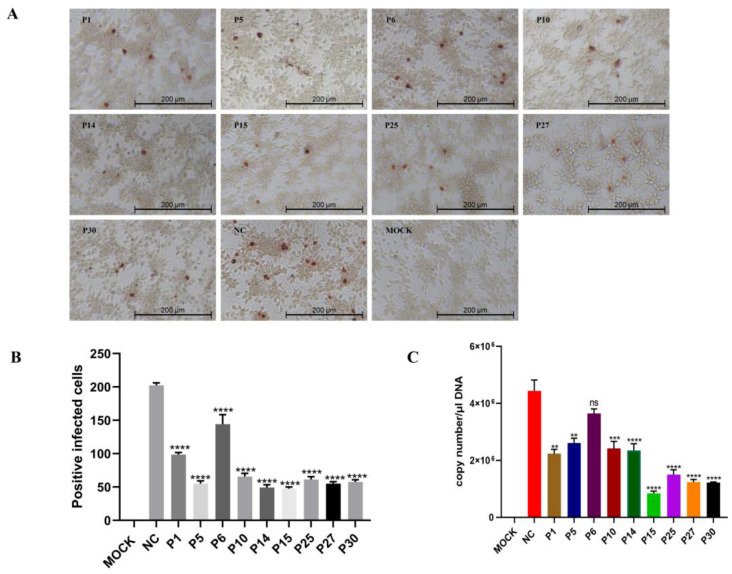
Screening of the antiviral effect of peptides against FAdV-4 infection in LMH cells. FAdV-4 virus (MOI = 0.01) was mixed with 200 μM peptides at 37 °C for 1 h and then inoculated onto near-confluent LMH cell monolayers at 37 °C. (**A**) The cells were fixed and incubated with a positive serum against FAdV-4 and HRP-conjugated rabbit anti-chicken IgG (H+L) at 37 °C. A total of nine peptides with antiviral effects were screened. Scale bars: 200 μm. (**B**) Statistical results of positive infected cells. The positive cell count was determined manually; three randomly chosen fields were observed under a microscope, and the average count of positive cells in the three fields was calculated to determine the total number of positive cells (100×). (**C**) Viral DNA was extracted from cells with DNA extraction kits, and then the FAdV-4 genome copies were measured using RT-qPCR. NC: negative control. Data represent means ± SEM from three independent experiments. **: *p* < 0.01; ***: *p* < 0.001; ****: *p* < 0.0001; ns: not significant.

To explore the antiviral effect of the 21 peptides against FAdV-4 infection, immunoperoxidase monolayer assays (IPMAs) were performed. Compared with the FAdV-4 control group that was not treated with peptides, the number of positive infected cells in the peptide (P1, P5, P6, P10, P14, P15, P25, P27, and P30) treatment groups were reduced, showing the antiviral effects of these peptides in a preliminary IPMA screening (Figure 4A). The number of positive cells was manually counted by selecting three fields (100×) of view under a microscope. The data were analyzed and presented in the form of a bar chart (Figure 4B). To further verify the antiviral effect, the genome copy number of FAdV-4 was measured. FAdV-4 virus (MOI = 0.01) was premixed with 200 μM peptides at 37 °C for 1 h, and then the mixture was inoculated into LMH cell monolayers. The genome copy number of FAdV-4 was detected with RT-qPCR at 24 h.p.i. (hours post-infection). The results show that the FAdV-4 copy number of the treatments with eight peptides (P1, P5, P10, P14, P15, P25, P27, and P30) reduced significantly in LMH cells compared with the untreated group and that P15 demonstrated the most obvious antiviral effect among the peptides (Figure 4C).

### 3.5. Interaction between the Peptides and the Knob Domain of the Fiber2 Protein

**Figure 5:**
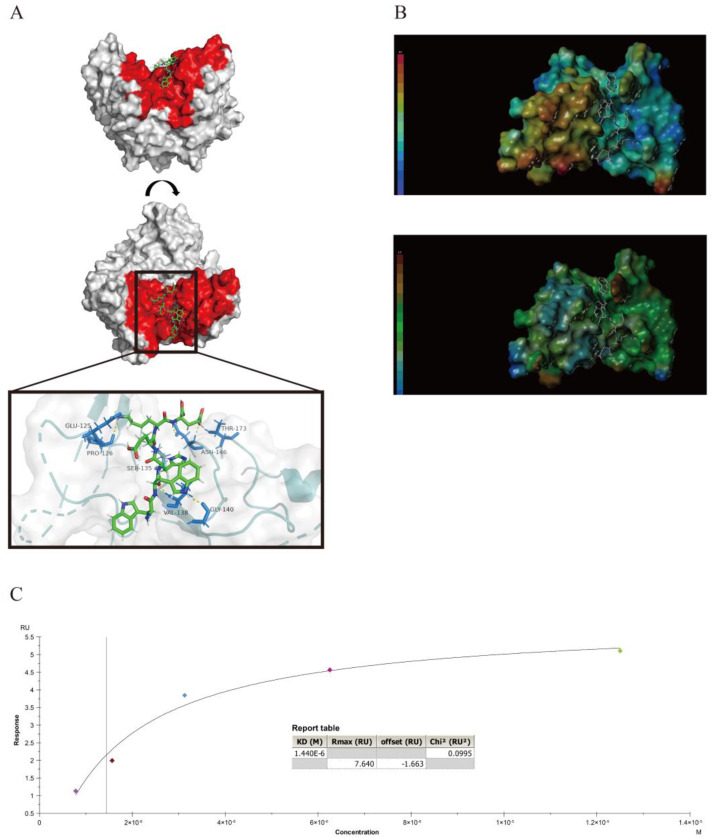
Interaction between the peptides and the knob domain of Fiber2. (**A**) P15 docked with the knob domain of the Fiber2 protein, and the hydrogen bonds of the interaction between them are shown with yellow dotted lines. The amino acids of the knob domain of the Fiber2 protein, which formed hydrogen bonds with the docking peptides, are colored in marine blue. The docking active pocket of the protein is colored in red. The analyses were carried out using Pymol software, version 4.6.0 (DeLano Scientific LLC company, Palo Alto, CA, USA). (**B**) The charge interactions (top figure) and hydrophobic forces (bottom figure) between P15 and the active site of the knob domain of the Fiber2 protein were analyzed using SYBYL-X 2.1.1 (Tripos, St. Louis, MI, USA). P15 and the knob domain of the Fiber2 protein are shown as a surface and ribbon model, respectively. The electrostatic potential ranges from red (most positive) to purple (most negative). The lipophilic potential ranges from brown (lipophilic) to blue (hydrophilic). (**C**) The real-time binding and fitting curve between the purified Fiber2 trimer protein and P15 was performed with SPR (Biacore X100, General Electric Company, Fairfield, CT, USA). The KD value was fitted and calculated with an appropriate model using Biacore X100 evaluation software, version 2.0.2 (General Electric Company, Fairfield, CT, USA).

To analyze the interactions between the peptides and the knob domain of the Fiber2 protein, the View/Surfaces and Ribbons/Create/MOLCAD module in the SYBYL-X 2.1.1 software and Pymol software were used to complete computer virtual analyses. The hydrogen bonds (yellow dotted lines) between P15 and the knob domain of the FAdV-4 Fiber2 are shown in Figure 5A, and the docking figures of other peptides are shown in Figure S4. The key amino acids at the binding site between P15 and the knob domain of the FAdV-4 Fiber2 were Glu-125, Pro-126, Ser-135, Val-138, Gly-140, Asn-146, and Thr-173. These hydrogen bonds contributed considerably to the interaction between P15 and the protein. The electrostatic interaction and hydrophobic interaction between P15 and the active site of the knob domain of the FAdV-4 Fiber2 protein are shown in Figure 5B. With the presence of oppositely charged residues on the interaction surface, the peptide and the protein may be attracted to each other because of electrostatic interactions. Therefore, the electrostatic interaction may also play an important role in the binding of P15 to the protein. The hydrophobic force may not be the dominant force, as the surface between P15 and the protein was not lipophilic or hydrophilic, as analyzed with SYBYL-X 2.1.1. Furthermore, surface plasmon resonance (SPR) assays were executed to determine the affinity between the nine peptides (P1, P5, P6, P10, P14, P15, P25, P27, and P30) and the knob domain of the Fiber2 protein. Meanwhile, the OVA was chosen as the unrelated protein, and the interaction with P15 was also determined to evaluate the selectivity interaction between P15 and the knob domain of Fiber2. As shown in Figure S5A,B, the results show that the response level of OVA–P15 was between 0 and 4 RU, which is similar to the OVA–HBS buffer. Thus, these responses seem to be meaningless signals. Additionally, the affinity fit was not able to obtain a rational result (Figure S5C). In contrast, the KD value between P15 and the knob domain of the Fiber2 protein was 1.44 μM (Figure 5C). The results of the other peptides are shown in Table 4. The KD values of the other eight peptides with the knob domain of the Fiber2 protein ranged from 0.57 to 23.97 μM, suggesting that the binding affinities of the peptides to the protein were strong.

### 3.6. Cytotoxicity of P15 in LMH Cells

**Figure 6:**
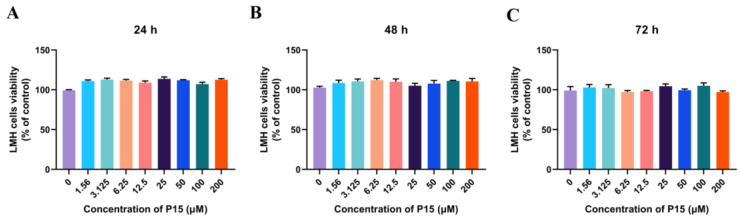
Cytotoxicity of P15 in LMH cells at different times. CCK-8 assays were used to detect the cytotoxicity of P15 to LMH cells at different concentrations after 24 h (**A**), 48 h (**B**), and 72 h (**C**) of incubation. Data represent means ± SEM from three independent experiments.

To analyze the effects of P15 at different concentrations on the vitality of LMH cells at different time points, cytotoxicity assays were performed using a CCK-8 assay. The LMH cells were treated with P15 at increasing concentrations (0, 1.56, 3.125, 6.25, 12.5, 25, 50, 100, and 200 μM), and the cytotoxicity was evaluated with CCK-8 after 24, 48, and 72 h of incubation. The results suggest that the treatment of P15 with different concentrations had no cytotoxic effect on the LMH cells after incubation for up to 72 h, compared with the untreated group (Figure 6).

### 3.7. P15 Inhibits the Infectivity of FAdV-4 In Vitro

**Figure 7:**
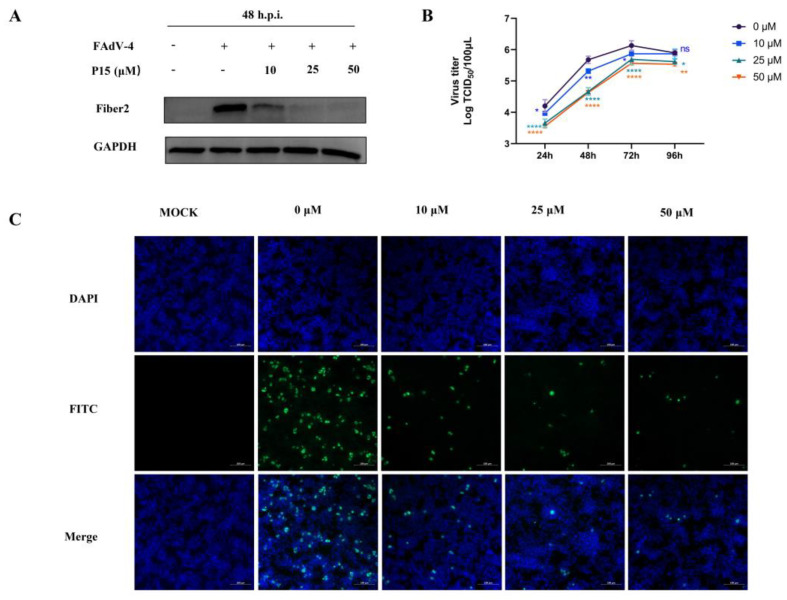
Impact of P15 on FAdV-4 infection in vitro. LMH cells were inoculated with a mixture of FAdV-4 virus (MOI = 0.01) and P15. (**A**) Fiber2 and GAPDH were assessed with Western blot analysis at 48 h.p.i. (**B**) TCID_50_ at various time points was calculated according to the Reed–Muench method. (**C**) IFA detection of FAdV-4-infected cells using mAb 5A5 and FITC-conjugated goat anti-mouse IgG antibodies. Scale bars: 100 μm. Data represent means ± SEM from three independent experiments. ns: not significant; *: *p* < 0.05; **: *p* < 0.01; ****: *p* < 0.0001.

To further validate the anti-FAdV-4 effect of P15, we analyzed the FAdV-4 Fiber2 protein expression and virus proliferation at different time points. FAdV-4 virus (MOI = 0.01) was premixed with peptides at 37 °C for 1 h, and then the mixture was inoculated onto LMH cell monolayers at 37 °C for 1 h. The cells were maintained in fresh DMEM containing 2% FBS without peptides. The expression level of the Fiber2 protein at 48 h.p.i. was measured using Western blot. As shown in Figure 7A, the Fiber2 protein expression in every P15-treated group (10, 25, and 50 μM) was reduced compared with the untreated group. The viral titers of FAdV-4 were significantly decreased in the P15 treatment group compared with the untreated group at 24, 48, 72, and 96 h (Figure 7B). The results indicate that viral proliferation was inhibited in a dose-dependent manner during the P15 treatment. Moreover, the results are consistent with the results of the immunofluorescence assays (IFAs) (Figure 7C). Taken together, P15 showed a pronounced antiviral effect against FAdV-4 infection. 

## 4. Discussion

FAdV-4 is the predominant etiological agent of HHS, which has caused a heavy economic burden on the poultry industry. There has been abundant research on developing a vaccine to prevent the disease but almost no research on the development of antiviral drugs [47]. Therefore, the development of an effective antiviral agent for preventing FAdV-4 infection is urgently needed. In this study, the C-terminal knob domain of the FAdV-4 Fiber2 protein was expressed and purified, protein with a high purity was crystallized, and its structure (PDB ID: 7W83) was determined. On the basis of the structure obtained, affinity peptides that specifically targeted the knob domain of the Fiber2 protein were designed and characterized. A total of eight peptides with potential antiviral activity were screened using IPMA and RT-qPCR. Treatment with P15 significantly reduced the levels of Fiber2 protein expression and viral titers during FAdV-4 infection, as determined with Western blot, IFA, and viral titer experiments. Therefore, P15 (WWHEKE) was confirmed to have an antiviral effect on FAdV-4 in LMH cells in vitro.

Peptides can effectively bind to the active site of a target protein to play a certain functional role with the help of precise design [45,48]. Peptides designed with molecular docking technology and computer virtual screening technology can be more accurately targeted to a protein site. Currently, commonly used high-throughput screening methods include phage display technology, mRNA display technology, combinatorial chemistry technology, and computer virtual screening technology [49]. Compared with other high-throughput screening methods, computer virtual screening technology has many incomparable advantages, such as a lower cost and shorter time for development [50,51,52]. The programs for the docking of peptide ligands with proteins include AutoDock vina [53], Glide [54], GOLD [55], Surflex-Dock [56], and GalaxyPepDock [57]. Surflex-Dock represents an advance in flexible molecular docking. There was a study showing that Surflex-Dock performed as well in terms of Root-Mean-Square Deviation (RMSD) accuracy for docked ligands and docking speed compared with the best methods in each category, and Surflex-Dock was significantly more accurate in its scoring with a 5 to 10-fold lower false positive rate than other methods for an equivalent true positive rate [58]. However, considering the complexity of the actual docking situation, it may be necessary to try different docking methods to find the best method for each protein, and we chose the most appropriate method in theory in this study. The same situation applied to the selection of the docking active pocket. The receptors of most adenoviruses including HAdV37 [59], HAdV-D26 [60], HAdV-5 [61], and CAV-2 [62] have been revealed, and the fiber-receptor complex structure has been determined [63,64]. It has been shown that the C-terminal domain interacts with the receptors [46,64,65]. However, research on the receptors’ interaction with FAdV-4 Fiber2 has not been reported yet. Considering that other adenoviruses usually interact with the receptor through the knob domain, we selected this C-terminal knob domain as the docking pocket, which is exposed to the outside and more likely to interact with other molecules. We selected most of the regions at the top of the trimer and the region between the two chains as targets, which represents a choice for exploring the possibility of different regions of the knob domain of the FAdV-4 Fiber2. Other locations are also potential sites. The optimal results indicate that our docking strategy was suitable and effective and provide ideas for the design of antiviral peptides against the infection of FAdV-4 and other diseases. Additionally, we acknowledge that there is no direct correlation between affinity and antiviral activity based on our experiment results. Furthermore, some peptides used in the study, such as P22, exhibited affinity to the Fiber2 protein but had no antiviral effects (Figure S6). Therefore, we infer that the antiviral effect of the peptide is not only related to the strength of affinity but also to the target site.

Our work provides the crystal structure of the C-terminal knob domain of the FAdV-4 Fiber2 protein for the first time. Furthermore, a structural characterization using gel-filtration chromatography and AUC revealed a trimer form of the protein. The crystal structure of the knob domain of the FAdV-4 Fiber2 protein showed that the asymmetric unit contained two monomers, which could form two trimers by generating a symmetry mate. Each monomer formed an anti-parallel β-sandwich with a topology similar to other known structures of knob domains of adenovirus fiber proteins. As previously reported by Zhang et al. [32], changes in the tail and knob regions of the Fiber2 resulted in virulence differences in the strains. Thus, the crystal structure of the C-terminal knob domain of the Fiber2 may provide a structural basis for studying the differences in virulence among various strains. Treatment with P15 significantly inhibited virus proliferation, probably through the binding of the peptide to the C-terminal knob domain of the FAdV-4 Fiber2 protein. The binding site may be of great significance for studying the effect of Fiber2 on FAdV-4 proliferation.

Though P15 significantly inhibited the proliferation of FAdV-4 in LMH cells, which was revealed with IPMA, RT-qPCR, Western blot, IFA, and viral titer experiments, the inhibition mechanism of P15 is still unknown. Many antiviral drugs work by blocking one or more of the virus cycle steps, including attachment to target cells [66], entry [67], replication [68,69], and release [70]; therefore, identification of the specific stages in which P15 performs antiviral activity would be helpful for investigating the inhibition mechanism of P15. Moreover, in vivo experiments to evaluate the antiviral effect of P15 are needed to facilitate a more comprehensive assessment of the antiviral potential of P15.

## Figures and Tables

**Table 1:** Crystallographic data and refinement statistics.

Crystal	Knob Domain of FAdV-4 Fiber2
**Data Collection**	
Space group	P63
Cell dimensions (length Å; angle °)	a = 60.34, b = 60.34, c = 192.60; α = β = 90, γ = 120
Resolution (Å)	50.48–1.29 (1.32–1.29) ^b^
R_merge_ (%) ^a^	9.1 (77.8) ^b^
Unique reflections	98,150 (6455) ^b^
Completeness (%)	98.8 (88.3) ^b^
<I/σ(I)>	16.8 (2.0) ^b^
Redundancy	17.3 (6.4) ^b^
**Refinement**	
Resolution (Å)	50.48–1.29 (1.32–1.29) ^b^
R_work_/R_free_ (%)	18.5/22.1
RMSD bond length (Å)/angle (°)	0.008/0.996
No. of molecules per asymmetric unit	2
No. of atoms	
Protein	3004
Water	782
Average B factors (Å^2^)	16.56
Ramachandran plot, residues in (%)	
Most favored regions	96.7
Additional allowed regions	2.5
Disallowed regions	0.8
**PDB ID**	7W83

^a^ R_merge_ = Σ(|I − < I >|)/Σ(I), where I is the observed intensity and < I > is the average intensity of all measured observations equivalent to reflection I. ^b^ Numbers in parentheses represent values in the highest-resolution shell.

**Table 2:** Primers used for RT-qPCR in this study.

Primer Name	Sequence (5’-3’)	Product Size/bp
FAdV-4 Hexon F	CGAGGACTACGACGATTA	95
FAdV-4 Hexon R	CGTGATACAGCAGGTTAATG	

**Table 3:** Sequences and total score values of peptides.

Peptide No.	Sequence	Total Score
P1	QWKKHI	16.3328
P2	MQKKWR	15.2680
P3	QWKKWW	14.8531
P4	QWKWMW	14.7913
P5	QWKIEK	14.7873
P6	QWKIHR	14.7709
P7	MQKEWQ	14.5475
P8	WWHQWD	14.5184
P9	WWHWEF	14.4116
P10	QWKNCK	14.3470
P11	WWHLPV	14.3400
P12	WWHEWQ	14.3068
P13	WWHEWG	14.2490
P14	QWKARV	14.1350
P15	WWHEKE	14.0258
P16	WWHSYT	14.0035
P17	QWKFEY	14.0002
P18	WWHHHC	13.9934
P19	WWHVEK	13.9661
P20	MQKRWK	13.9108
P21	QWKQKW	13.9061
P22	QWKAFG	13.8920
P23	WWHGHM	13.8756
P24	MQKADM	13.3874
P25	MQKHEW	13.2421
P26	MQKYTY	13.1730
P27	MQKHCG	13.1302
P28	MQKLWW	13.0898
P29	MQKQWI	12.9858
P30	MQKKFI	12.9694

**Table 4:** The KD values of the eight peptides with the knob domain of the Fiber2 protein.

Peptide No.	KD (M)
P1	6.161 × 10^−6^
P5	1.199 × 10^−6^
P6	2.779 × 10^−6^
P10	1.021 × 10^−6^
P14	2.397 × 10^−5^
P25	1.367 × 10^−6^
P27	5.725 × 10^−7^
P30	9.070 × 10^−7^

## Data Availability

Not applicable.

## Supplementary Materials

The following supporting information can be downloaded at: https://www.mdpi.com/article/10.3390/v15040821/s1, Figure S1: The crystal picture of FAdV-4 Fiber2 knob. Figure S2: The sequences of the docking active pocket. The sequences of docking active pockets are colored in yellow. Figure S3: Cytotoxicity of P3, P8, P9, P11, P12, P18, P19, P23, and P29 in LMH cells at a concentration of 200 μM for 24 h. Figure S4: The docking of peptides (P1, P5, P6, P10, P14, P25, P27, and P30) to the knob domain of the FAdV-4 Fiber2 protein. The hydrogen bonds between the peptides (P1, P5, P6, P10, P14, P25, P27, and P30) and the knob domain of the FAdV-4 Fiber2 protein are shown as a yellow dotted line. The amino acids of the knob domain of the Fiber2 protein that formed hydrogen bonds with the docking peptides are colored in marine blue. Figure S5: The response of P15 to OVA and HBS buffer and the affinity fit result. (A) The response level of OVA–P15 was between 0 and 4 RU. (B) The HBS buffer was almost the same with OVA–P15. (C) The affinity fit of OVA–P15 was not able to obtain a rational result. Figure S6: (A) The real-time binding and fitting curve between purified Fiber2/trimer protein and P22 was performed with SPR assay. The KD value was fitted and calculated with the appropriate model using Biacore X100 Evaluation software, version 2.0.2 (General Electric Company, Fairfield, CT, USA). (B) The IPMA image of P22. Scale bars: 200 μm.
