# Supplementary material for: Design and Identification of a Novel Antiviral Affinity Peptide against Fowl Adenovirus Serotype 4 (FAdV-4) by Targeting Fiber2 Protein
Source: Viruses. 2023 Mar 23;15(4):821. doi: 10.3390/v15040821 (PMC10146638; doi:10.3390/v15040821)
Supplement: Supplementary file 1 [file viruses-15-00821-s001.zip › viruses-2267769 supplementary figures.pdf]

Article

Design and Identification of a Novel Antiviral Affinity Peptide Against Fowl Adenovirus Serotype 4 (FAdV-4) by Targeting Fiber2 Protein

Supplementary files

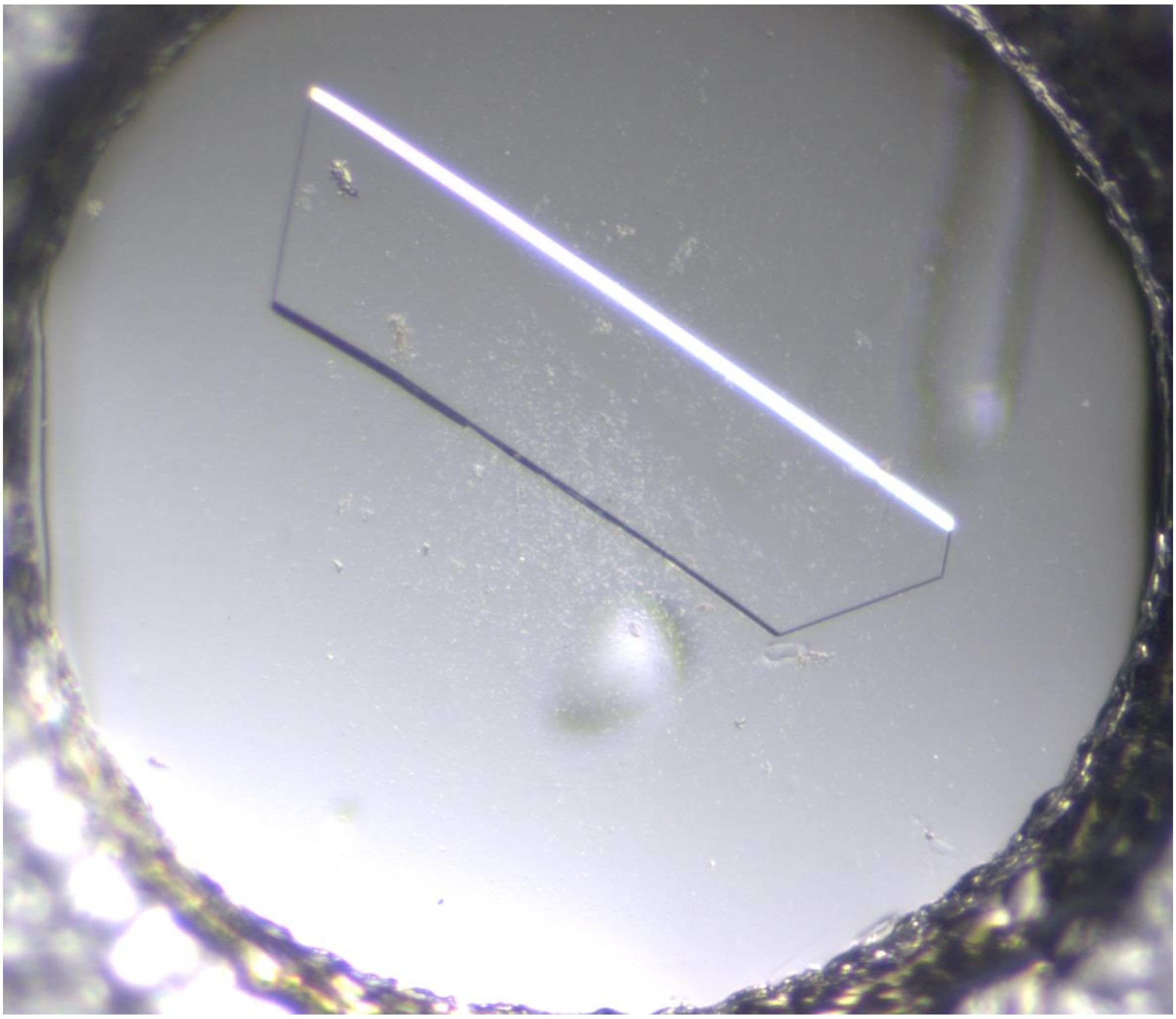

Figure S1: The crystal picture of FAdV-4 Fiber2 knob.

Chain A
VSTPIATFVSGSPSLNTYNATTVNSSANAFSCAYYLQQWNIQGLLVTSLYLK
LDSATMGNRPGDLNSANAKWFTFWVSAYLQQCNPSGIQAGTVSPSTATLT
DFEPMANRSVTSPWTYSANGYYEPSIGEFQVFSPVVTGAWNPGNIGIRVLP
VPVSASGERYTLLCYSLQCTNASIFNPNNSGTMIVGPVLYSCPAASLP

Chain B
VSTPIATFVSGSPSLNTYNATTVNSSANAFSCAYYLQQWNIQGLLVTSLYLK
LDSATMGNRPGDLNSANAKWFTFWVSAYLQQCNPSGIQAGTVSPSTATLT
DFEPMANRSVTSPWTYSANGYYEPSIGEFQVFSPVVTGAWNPGNIGIRVLP
VPVSASGERYTLLCYSLQCTNASIFNPNNSGTMIVGPVLYSCPAASLP

Figure S2: The sequences of the docking active pocket. The sequences of docking active pockets are colored in yellow.

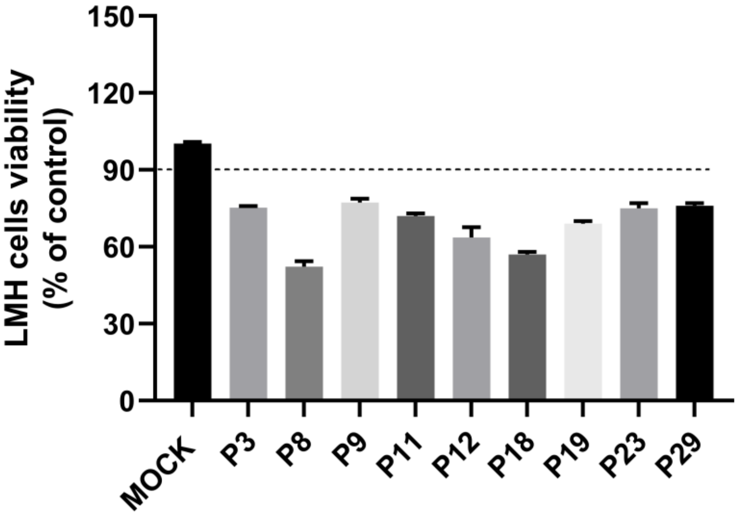

Figure S3: Cytotoxicity of P3, P8, P9, P11, P12, P18, P19, P23, and P29 in LMH cells at a concentration of 200 μM for 24 h.

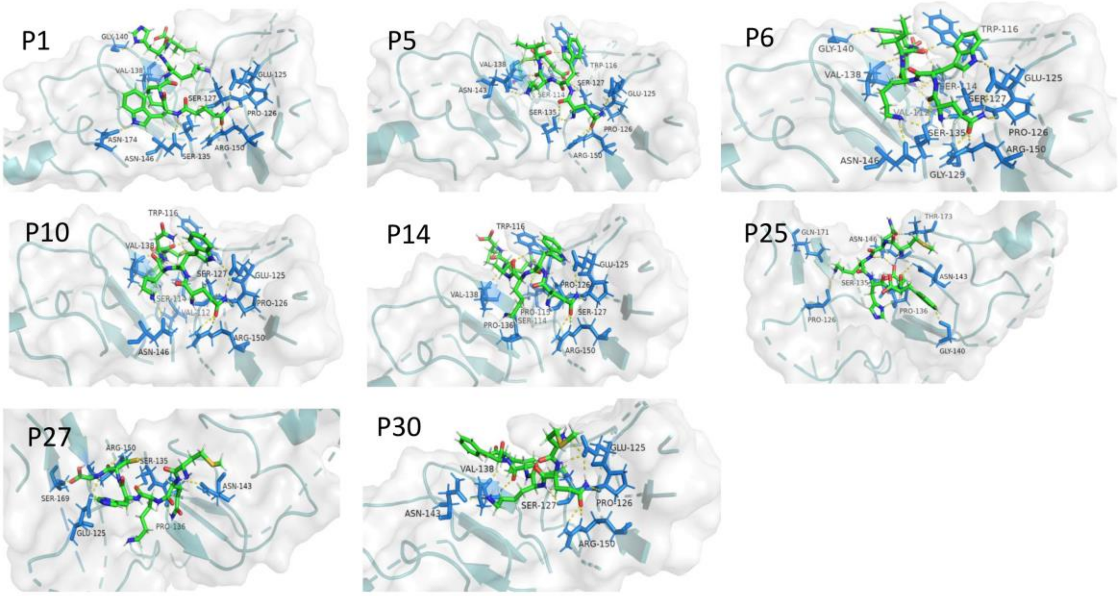

Figure S4: The docking of peptides (P1, P5, P6, P10, P14, P25, P27, and P30) to the knob domain of the FAdV-4 Fiber2 protein. The hydrogen bonds between the peptides (P1, P5, P6, P10, P14, P25, P27, and P30) and the knob domain of the FAdV-4 Fiber2 protein are shown as a yellow dotted line. The amino acids of the knob domain of the Fiber2 protein that formed hydrogen bonds with the docking peptides are colored in marine blue.

A

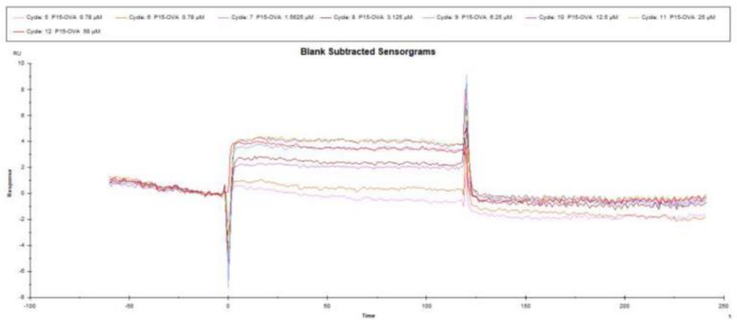

B

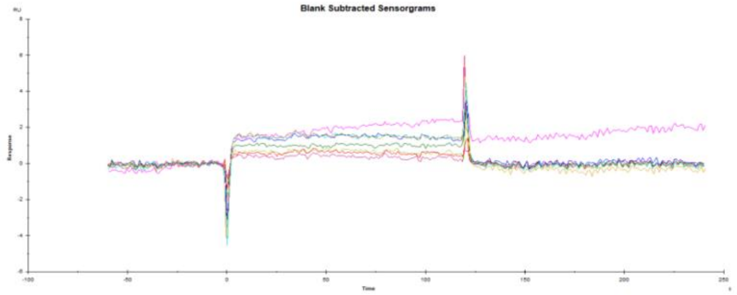

C

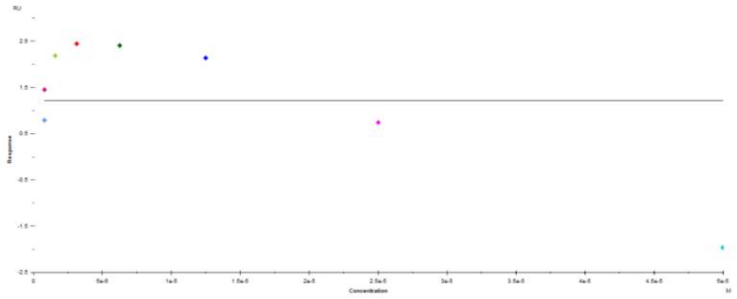

Figure S5: The response of P15 to OVA and HBS buffer and the affinity fit result. (A) The response level of OVA–P15 was between 0 and 4 RU. (B) The HBS buffer was almost the same with OVA–P15. (C) The affinity fit of OVA–P15 was not able to obtain a rational result.

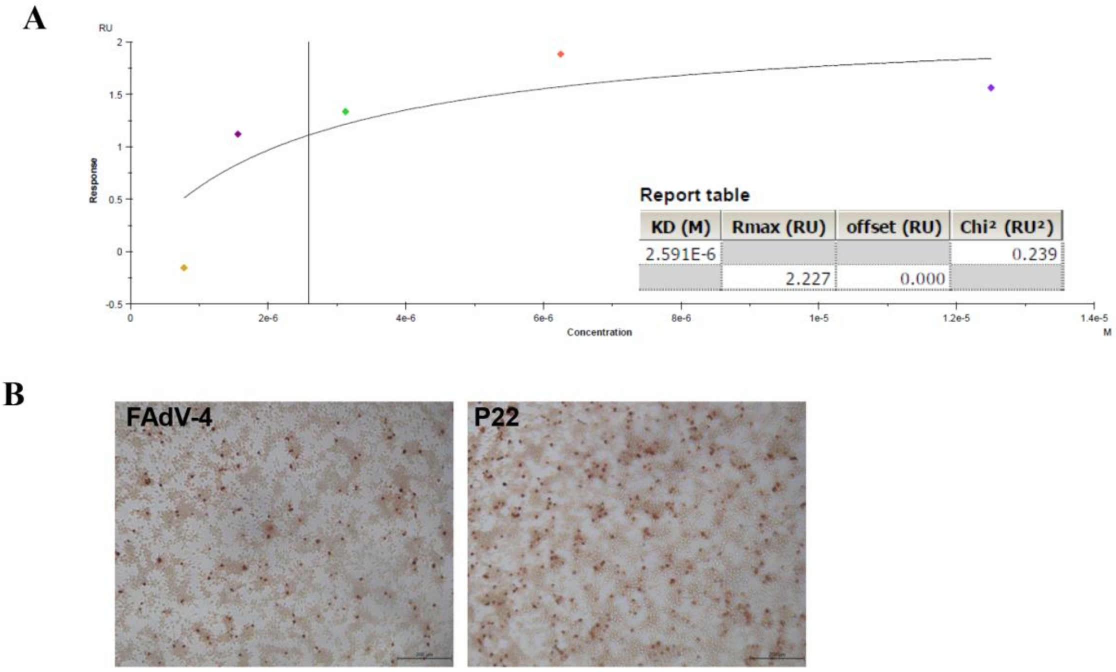

Figure S6: (A) The real-time binding and fitting curve between purified Fiber2/trimer protein and P22 was performed with SPR assay. The KD value was fitted and calculated with the appropriate model using Biacore X100 Evaluation software, version 2.0.2 (General Electric Company, Fairfield, Connecticut, USA). (B) The IPMA image of P22. Scale bars: 200 μm.
